# Self-moving multi-sensor AI-based robotic technology for road crack inspection

**DOI:** 10.3389/frobt.2026.1811691

**Published:** 2026-06-23

**Authors:** Hafsa Matich, Najia Ait Hammou, Hajar Mousannif, Abdellah El Aissaoui

**Affiliations:** 1 LISI Laboratory, Computer Science Department, Faculty of Science Semlalia, Cadi Ayyad University, Marrakesh, Morocco; 2 Laboratory of Agricultural Machinery and Energy, National Institute of Agronomic Research, CRRAS, Settat, Morocco

**Keywords:** autonomous road inspection, deep learning, mobi-A4Net, pavement crack characterization, robotic-AI systems

## Abstract

Human inspectors conducting road inspections face both heavy physical demands and subjective judgment, which can affect the accuracy of road surface evaluations. This study introduces Mobi-A4Net, an affordable robotic-AI system for automatic road crack detection and assessment, addressing limitations of costly automated systems and lack of integration with existing technologies. The system focuses on an unmanned ground vehicle (UGV) platform for detailed inspection work. The Mobile Adaptive Attention Aggregation Network forms the core of the system, implementing a compact deep-learning model with multi-scale attention mechanisms to identify thin, low-contrast cracks within complex surface patterns. Advanced image-processing techniques, including Medial Axis Transform (MAT) skeletonization, enable real-time measurement of crack length, width, and orientation. Experimental results show that Mobi-A4Net achieves 99.7% detection accuracy, a recall rate of 98.8%, and a mean Intersection over Union (mIoU) of 95.4%. With 1.85 million parameters and an inference speed of 9.6 milliseconds per image, the system is suitable for real-time operation on embedded UGV platforms.

## 1 Introduction

### 1.1 Problem definition

Road cracks serve as initial signs indicating that pavement and structural elements begin to deteriorate, requiring prompt identification to prevent further damage, expensive repair work, and safety risks. Traditional inspection methods rely on manual visual assessments by field workers, which require substantial labor and time and produce results dependent on personal judgment. These limitations make it difficult to evaluate crack conditions accurately, particularly over extensive transportation networks (Zhu et al., 2019; Dai et al., 2025; Kowsalya, 2023). Automated systems are needed to provide reliable, repeatable inspections while reducing human workload.

The new developments in robotics and artificial intelligence create opportunities to transform pavement inspection operations. In this study, we focus exclusively on unmanned ground vehicles (UGVs) for detailed, high-resolution inspections of road cracks. Ground robots provide precise imaging capabilities for accurate crack measurement through high-resolution cameras. The robotic system, combined with deep learning-based detection models, enables immediate crack detection and quantitative assessment, improving inspection efficiency and coverage beyond standard manual methods (Yıldızlı et al., 2025; Kim et al., 2024).

Automated crack monitoring systems have demonstrated their capacity to identify cracks with over 90% accuracy while reducing inspection duration in real-world applications (Song et al., 2025; Dong et al., 2022). However, existing technology has not yet produced dependable crack detection systems that operate robustly in outdoor conditions, where surfaces exhibit variable textures, lighting changes, and cracks of diverse widths and lengths. Environmental noise, shadows, occlusions, and surface contaminants create challenges for feature extraction and degrade algorithm performance. Many current systems operate as separate entities, either using sensing hardware or detection algorithms, without integrating mobility, perception, and AI processing (Dai et al., 2025).

### 1.2 Related works

The development of Unmanned Ground Vehicle systems for automated crack inspection has progressed through sensor improvements and better mobility systems and deep learning technological advancements. UGVs enable precise navigation through difficult terrains which permits engineers to perform detailed inspections and monitor structural health at close distances.

Mobile UGV platforms for structural crack detection. The development of the real-time mobile robotic system in their study uses RGB-D cameras and LiDAR sensors according to Ogun et al. (2026). The system employs a two-stage deep learning framework which uses U-Net for crack detection and Pix2Pix to boost detection resolution. The system successfully navigated construction sites by mapping micro-cracks which range between 0.5 mm and showed effectiveness in difficult-to-navigate areas with obstructed visibility. The UGV system which Nitta et al. (2023) developed executes indoor wall inspections through high-resolution imaging to build orthoimages while using YOLO-v7 for crack detection. The UGV system enables wall inspections which require less human activity to examine large wall sections. The UGV platform from Yang et al. (2025) integrates both a 2-DoF manipulator and stereo vision sensors to perform crack measurement and monitoring tasks on concrete structures. The manipulator enables users to inspect from close range while the ground platform maintains its full movement capabilities.

Autonomous real-time inspection systems. The UGV-based pavement inspection system from Khan et al. (2023) uses autonomous navigation together with real-time imaging and deep learning for crack detection. The system can operate in different lighting conditions which includes both shadowy and bright environments while achieving accurate detection across various pavement types. Mei et al. (2022) presented ROADS as a UGV prototype which examines pavement damage through multispectral imaging technology. The ROADS system proved its capability to map extensive urban areas while creating precise crack maps for upkeep operations. The researchers Zia et al. (2025) achieved a major advancement in robotic inspection by connecting digital twins to their system which enables automatic structural crack detection and ongoing damage assessment. The system used predictive modeling to create parallel simulations of crack development with actual measurement data from the field. Song et al. (2025) created a dual-branch learning framework which uses visual and geometric data from a robotic sensing platform to detect road cracks in real time under difficult noise and low-contrast circumstances.

Advanced vision and deep learning approaches. The intelligent vision-based pipeline developed by Matich and Mousannif (2026) enables automated defect detection through its use of adaptive contrast enhancement and attention-based deep neural networks which boost detection accuracy for shadows and occlusions and surface irregularities. The 3D vision system developed by Hu et al. (2024) creates accurate 3D profiles of structural cracks through its use of LiDAR and structured-light sensors. The hierarchical deep learning model designed by Deng et al. (2026) enables automated health monitoring of road infrastructure through UGVs by improving performance across various environmental conditions and surface materials. The complete evaluation of autonomous robotic systems which Dai et al. (2025) provided focuses on UGV platforms which combine perception and cognition with autonomous civil infrastructure monitoring capabilities. The research by Chen et al. (2024) about UAV-assisted systems provides frameworks for multi-modal data fusion which will support forthcoming hybrid UAV–UGV systems.

The research collectively demonstrates how UGVs enable organizations to implement automated crack inspection systems which provide benefits through their capacity to deliver high-resolution images and move across various terrains while combining information from different sensor types. The researchers need to enhance operational systems because they currently struggle with environmental conditions and operational areas and real-time maintenance decision-making. The combination of advanced deep learning algorithms with sensor fusion and autonomous navigation continues to drive the evolution of UGV-based structural health monitoring systems (Genova et al., 2024).

### 1.3 Objective and contributions

To fill the research gaps, this paper proposes an integrated UGV-based robotic-AI system for detecting, identifying, and assessing cracks on road surfaces. The proposed system comprises three interdependent components: (i) UGV platforms for flexible and precise data acquisition, (ii) deep learning-based algorithms for crack detection, characterization, and classification, and (iii) autonomous navigation and data processing modules for real-time operation (Dai et al., 2025; Zhang et al., 2024; Recalde et al., 2024).

Within this framework, the proposed Mobi-A4Net performs a multi-stage crack analysis process. First, the system determines whether a crack is present in the captured image using Image Classification (crack/no crack). Second, it applies Semantic Segmentation to localize and highlight crack pixels. Third, the detected crack is categorized into structural types (horizontal, diagonal, or mixed). Finally, the segmentation output is used to extract quantitative measurements such as crack length and width, enabling a detailed assessment of crack severity.

The core objective of this research involves developing a robotic-AI framework which includes design and implementation and scaling processes to achieve better detection accuracy and operational efficiency and improved decision-making for road maintenance and infrastructure management. Amongst other contributory factors, the advancement of knowledge in this work lies in closing the technological gap between robotic sensing and AI-based crack analysis to demonstrate the synergistic potential of UGV inspection in forming the basis of intelligent, autonomous, and data-driven infrastructure monitoring. This way, the research advances practical integration of robotics and AI for the purposes of complementary tools improved efficiency, reliability, and safety of road inspection processes.

### 1.4 Outline

The paper maintains its structure through three main sections. The Materials and Methods section describes the system development process through its complete system development process which includes dataset preparation and image pre-processing and model training strategies and robotic navigation system development with real-time inference capabilities. The experimental results from Section 3 demonstrate their practical use while comparing their strengths to existing methods and their remaining challenges. The paper ends in Section 4 which presents its key findings before describing future research plans for large-scale deployment and autonomous decision support and improved multi-robot collaboration in intelligent road infrastructure monitoring.

## 2 Materials and methods

The research project combines an autonomous navigation system with real-time road surface and infrastructure monitoring capabilities. The UGV system conducts field assessments to locate road blockages and detect pavement deterioration. The system generates essential information which local transportation departments and municipalities and airport administrators can use.

The platform main purpose exists to perform large-scale road network mapping and infrastructure asset surveying activities. The system operates through its dedicated purpose to conduct road surface and bridge deck investigations while detecting all visible cracking. Image processing techniques are used to calculate the crack dimensions which include both width and length according to Lei et al., 2018; Dorafshan et al., 2019. The UGV uses its high-resolution imaging sensor together with image binarization methods to enhance crack detection capabilities. The method enables scientists to conduct crack analysis using ground inspection findings which produce precise and trustworthy pavement condition assessments.

### 2.1 Mechanical conceptual design

**FIGURE 1:**
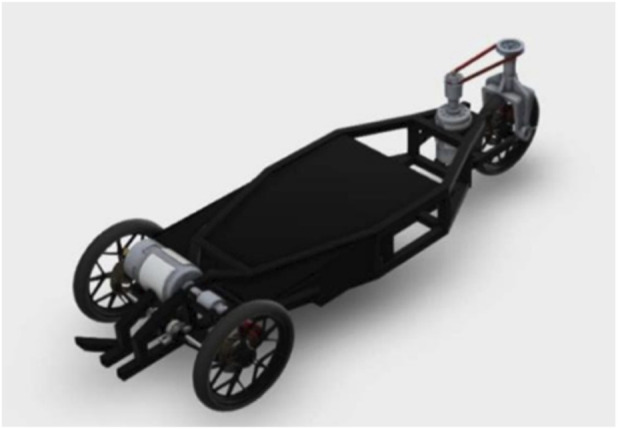
Tricycle robot design. Reproduced with permission from (El Farnane et al., 2022).

The mechanical conceptual design of the Mobi-A4Net system represents the initial stage of the design process (Figure 1), where the general forms and functional objectives of the platform are established. This phase establishes product performance requirements while determining user interactions which will create a system that delivers easy operation and performs its essential functions. The road crack detection requirements establish platform compatibility with all surface types which demands development of a durable physical system.

#### 2.1.1 Functional analysis and technical specification

The design process starts with a detailed functional analysis to establish complete system requirements. The procedure divides the complex goal of autonomous inspection into multiple easier-to-handle sub-functions which enables precise PDS development. The analysis process identified three core technical functions which include:

- Autonomous Locomotion: Powering the drive system through high-capacity batteries and DC motors.
- Precision Navigation: Facilitating movement through linear segments and complex maneuvers using a holonomic 4WD/4 W S (four-wheel drive and four-wheel steer) configuration.
- Real-Time Localization: Identifying the robot’s spatial position *via* a GPS module to ensure accurate mapping of detected features.
- Feature Perception: Utilizing a specialized vision system to detect and identify discontinuities on the road surface.

#### 2.1.2 Physical architecture and component integration

The architectural plan establishes a modular physical system which operates from an advanced onboard computing core designed to withstand harsh conditions. The hardware system enables exteroceptive sensors and actuators to establish direct communication links while the system maintains its operational effectiveness and structural integrity.

- Chassis and Drive Train: The framework supports the fundamental mechanical and electrical components, featuring a holonomic wheel system that allows for independent steering and drive control. This setup is essential for maintaining the steady imaging trajectories required for high-resolution pavement analysis.
- Sensor Integration: The physical form is designed to house a Perception and Inspection suite (DSLR camera and vision-based controllers), a Guidance suite (navigation sensors, GPS, and Radar), and a Power Management suite (lithium batteries and DC/DC converters).
- Sensing Geometry: To optimize data acquisition, the primary inspection camera is mounted at a calculated height and angle—specifically 0.53 m above the ground with a 30° tilt—to capture a field of view necessary for identifying minute pavement defects.

The mechanical system design process uses this systematic method to connect technical requirements with physical system elements which enables the project to move into its subsequent design and building phase.

### 2.2 On-board electronic system design

**FIGURE 2:**
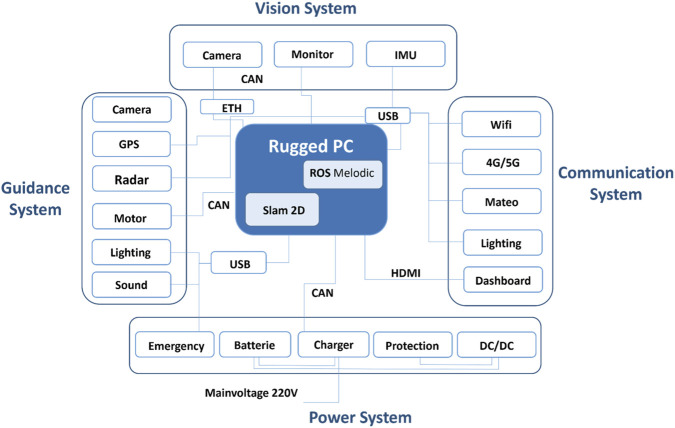
UGV system architecture (HW and SW).

The Mobi-A4Net system architectural design establishes a modular hierarchical framework which uses a rugged computing core to satisfy autonomous road inspection requirements. The design enables physical sensing hardware to interact with high-level AI processing units through an established robust connection. The robot requires exteroceptive sensors for obstacle detection and terrain tracking which leads to a hardware (HW) and software (SW) architecture that divides into four functional parts which connect through a central Rugged PC that operates Robot Operating System (ROS) and Slam modules (Figure 2).

#### 2.2.1 Vision and perception system

This module is basically responsible for high-resolution environment characterization and for the execution of sensing tasks.

- Camera: Captures real-time image streams to detect the position of objects (such as crops or road distress) in relation to the robot, ensuring correct positioning.
- Micro-controller: Acts as the “brain” of the vision system, processing information from perception sensors to generate control signals for the motors (Gridling and Weiss, 2007; Reverter, 2012; Ernst et al., 2002).
- Analysis Sensors: Includes a soil analysis sensor to transmit data on nitrogen and organic matter; in an inspection context, this component slot is dedicated to the specific sensors required for surface characterization (Nadporozhskaya et al., 2022).

#### 2.2.2 Guidance and spatial awareness system

This part ensures the robot maintains its trajectory while identifying hazards and documenting its location.

- Navigation Camera: A dedicated camera used specifically for autonomous guidance, obstacle detection, and row or lane determination.
- GPS: Provides critical spatial information, giving the system the ability to evaluate, document, and track its performance in relation to its global coordinates (Shen and Stopher, 2014; Turner et al., 2000).
- Radar: A remote sensing system that identifies stationary and moving obstacles by emitting electromagnetic waves to determine the position and speed of detected objects. It is noted as the most reliable method for obtaining environmental data regardless of lighting conditions. To detect the presence and determine the position and speed of objects

#### 2.2.3 Power and operational safety system

The electrical architecture is designed to support autonomous long-term operation and critical failure protection.

- Batteries: Accumulate electrical energy to supply drive motors and electronics with sufficient current to work effectively (Li et al., 2018).
- DC/DC Converter: Transforms the DC source from the battery to the specific voltage levels required by various electronic components (Dahale et al., 2017; Hossain et al., 2018).
- Charger Module: Transforms alternate voltage into direct voltage to recharge the electric accumulators (Ayob et al., 2014; Berrehil El Kattel et al., 2023).
- Protection System: An indispensable component that guards the power supply against over-current, over-voltage, and overheating (Gabbar et al., 2021; Winanta et al., 2023).
- Emergency Stop: A safety mechanism that causes an immediate cessation of any process by cutting power to the actuators (Stolt et al., 2011; Hu et al., 2024).

#### 2.2.4 Communication and telemetry system

This layer facilitates proactive management and real-time data sharing with users.

- WiFi Module: An interface that allows micro-controllers to connect to local networks and transfer data between different system elements (Hlaing et al., 2017; Mesquita et al., 2018).
- 4G/5G Modem: A connectivity box that links all onboard equipment to the internet for remote monitoring and data offloading (Barb and Otesteanu, 2020; Agiwal et al., 2021).
- Weather Station: Measures environmental parameters (air temperature, humidity, wind, etc*.*) to help optimize the robot’s actions and predict environmental influence on operations (Ioannou et al., 2021; Tenzin et al., 2017).
- Three-color Light: An industrial signal indicator used to visually signal the robot’s status, such as “start,” “stop,” or “fault”.
- Dashboard: A decision-making GUI that facilitates proactive management and evaluates the robot’s performance in real-time (Bach et al., 2022).

The software architecture establishes connections between hardware components through its behavioral design method. All controllers for tasks like goal-seeking and obstacle avoidance operate simultaneously, with their outputs (velocities and steering angles) being arbitrated by a PID (classical robotics) or a Mamdani-type fuzzy supervisor in complex systems. Each controller uses demand-based weighting to manage functional requirements, which allows the robot to travel towards its target while avoiding obstacles. Both controllers take as inputs distance and steering angle, while the outputs are the velocity and the front wheel angle. The specific software pipeline for vision-based tasks uses preprocessing (grayscale conversion and Gaussian blur) to prepare data for RANSAC-based line/object detection, then gathers it with a PID control which controls steering movements. The implementation of the RANSAC algorithm step is dedicated to the best and most precise line detection in noisy situations before pursuing crack detection (N. Ait hammou et al.).

### 2.3 System implementation

**FIGURE 3:**
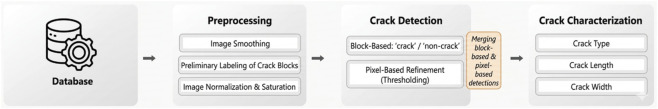
Crack detection image processing system architecture.

The crack detection module serves as the primary perception element of the autonomous navigation system because it detects pavement surface conditions through its analysis. The onboard vision sensor captures raw images which get transmitted to the embedded processing unit, where the system operates its image-processing and learning-based pipeline for real-time image analysis according to Figure 3. The module operates through two main components which include the model’s performance and the inference process that depends on training data quality and preprocessing methods. The following subsections present the dataset creation process, which includes ground-truth generation and image-processing pipeline development used to validate the proposed crack detection model.

#### 2.3.1 Dataset description and acquisition protocol

**FIGURE 4:**
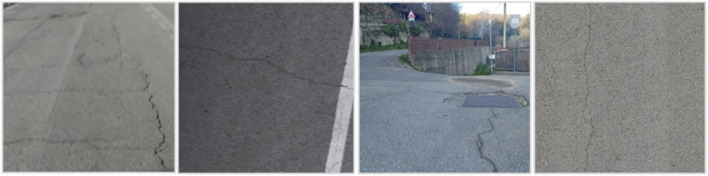
Sample images from the Crack dataset used in this study.

The crack database (Figure 4) used in this study consists of post-event inspection survey images collected after the Al Haouz earthquake, which were obtained from local authorities and field observations and public satellite imagery. The dataset is divided into two main folders which are named “filtered” and “unfiltered” and both folders have “pos” (positives) and “neg” (negatives) folders inside them. The unfiltered dataset contains all original images which have not been deleted yet while the filtered dataset includes resized JPG images which passed quality control before processing. The dataset handles the organization of data through two folders which contain cracked pavement images in the “neg” folder and normal pavement surfaces in the “pos” folder for supervised learning and evaluation purposes. The complete database contains approximately 300,000 images which serve as its entire collection.

Field acquisition of the subset used a Canon EOS 1100D DSLR camera which featured a 30 mm lens and 12.2 MP CMOS sensor and operated from a tripod to reduce framing distortion and parallax errors. The image capturing process used ambient diffuse lighting during early morning hours because this time prevents glare and shadow creation and color distortion of the captured images. The standard acquisition parameters were adopted (aperture f/8, shutter speed 1/125 s, ISO 100) to create uniform exposure and consistent results across all samples. The accepted images followed all acquisition restrictions which included maintaining a fixed camera-surface distance and stable illumination and frontal orientation and achieving sufficient spatial resolution to display fine-crack details. The acquisition process required traceability through documentation which showed all difficult situations that occurred with shadows and dust and surface irregularities. Geolocations were added to images while metadata were tagged with capture device details and lighting condition and viewing angle and surface context information to promote reproducibility and facilitate dataset interpretability.

#### 2.3.2 Ground truth mask generation and dataset split

Ground-truth crack masks were manually annotated by professional engineers who utilized image-editing software such as Photoshop and GIMP to delineate crack segments. After an independent verification process, the masks were forwarded to evaluate the segmentation methods developed and to kick-start an assessment of related performance in comparison to existing tools like CrackIT (Yeum and Dyke, 2015) and the MATLAB Image Processing Toolbox. The dataset, consisting of a total of 300,000 images, was split randomly into 80% training (240,000 images) and 20% testing (60,000 images), while ensuring that images from the same scene were not divided across subsets to prevent spatial bias and data leakage.

#### 2.3.3 Image-processing pipeline and preprocessing operations

Crack detection processes were based on a mixed image-processing and software-assisted workflow that can estimate crack length and type to provide quantitative structural assessment. The processing pipeline runs through database preparation, preprocessing, crack detection with pixel- and block-based strategies, and crack characterization; the preprocessing stage consists of the following.

- Light smoothing to remove background texture noise while maintaining the presence of fine cracks,
- Block labeling to avoid losing relevant regions in crack images during normalization and saturation correction,
- Intensity normalizing and saturation balance to minimize variability of illumination and suppress specular reflections.

In conjunction with contrast enhancement, these stages go a long way toward mitigating lens distortion and acquisition inconsistencies, in turn improving detection robustness across diverse image sources.

#### 2.3.4 Pixel-based and block-based crack detection strategy

The detection stage utilizes two complementary algorithms. The block-based method (Abdellatif et al., 2021) classifies image blocks as crack or non-crack with the help of a pattern-recognition technique, while a pixel-based method executes threshold-based segmentation of the preprocessed grayscale images. Connected-components analysis is performed on identified candidate crack blobs, followed by binary feature extraction of their geometry after correction against orientation, once preliminary crack-region localization has been executed. Histogram intensity analysis helps guide threshold selection so that segmentation boundaries tightly follow crack contours and thus enable accurate quantification. While efficient, these classical techniques may face performance challenges in real-time or extremely variable environmental conditions.

#### 2.3.5 Deep-learning-based detection using Mobi-A4Net

**FIGURE 5:**
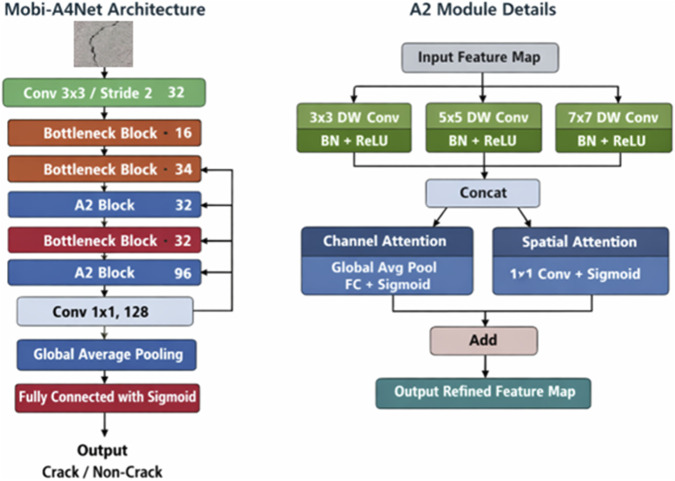
Overall architecture of the proposed Mobi-A4Net and detailed structure of the A2 module.

The existing compact architectures find it increasingly difficult to achieve the desired level of cracked-detection performance. This is owing to the inclinations of mobile and field operations toward the utmost constraints. The architecture is termed as Mobi-A4Net (Mobile Adaptive Attention Aggregation Network), which was designed to enhance crack-detection performance under such mobile and field-operational constraints. The network retains the MobileNet-style encoder design and introduces an adaptive multi-scale attention aggregation (A2) mechanism, which enhances feature saliency for thin and low-contrast cracks while controlling computational cost (Figure 5).

As described in Table 1, with about 1.85 million parameters and requiring approximately 120 MFLOPs for a 224 × 224 input image, Mobi-A4Net ranks among the ultra-lightweight vision backbones (Zhu et al., 2025), while still holding decent capacity in representation. Starting with a convolutional stem 3 × 3 (32 filters), the architecture is composed of inverted-residual-type bottleneck blocks in sequence, with expansion ratios from three to six and depthwise-separable convolution to induce efficient channel-expansion-compression without incurring too many computational costs.

**TABLE 1:** Sample analysis of road images for crack type classification and measurement.

Img	Detected	Ground truth	Type	Length (px)	Width (px)
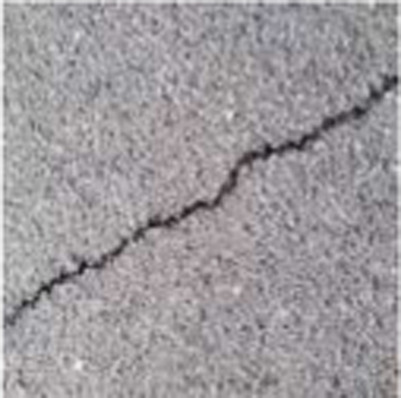	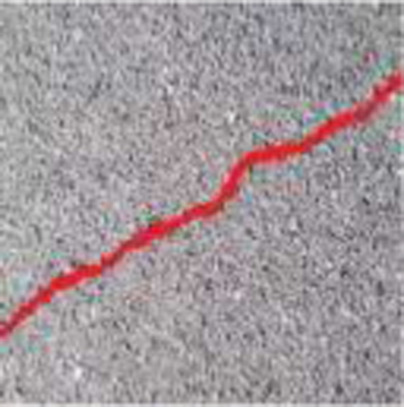	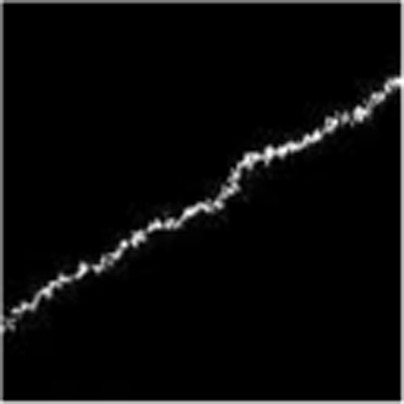	Diagonal (-61.85°)	2,313.88	400.10
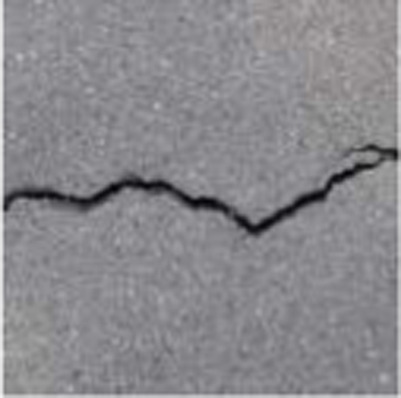	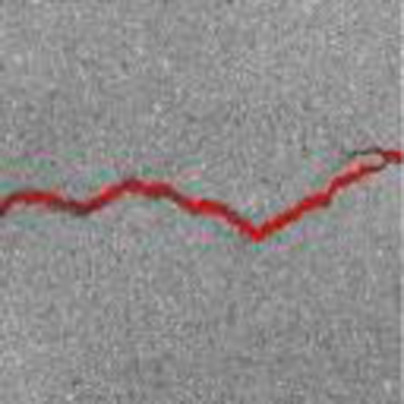	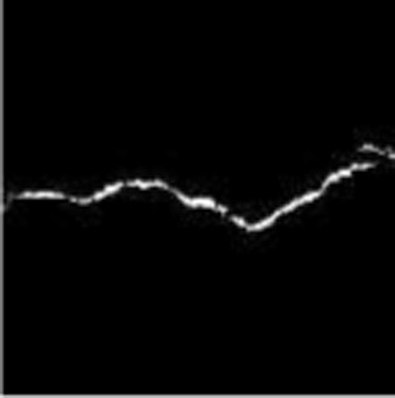	Mixed	2,267.71	340.32
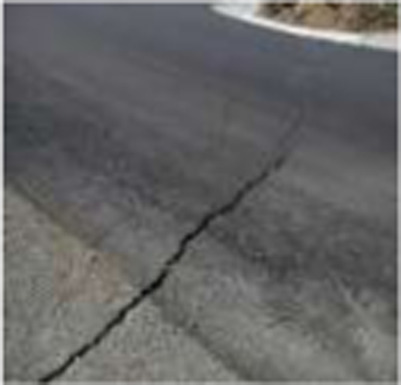	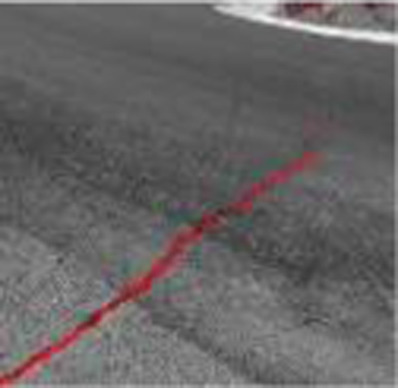	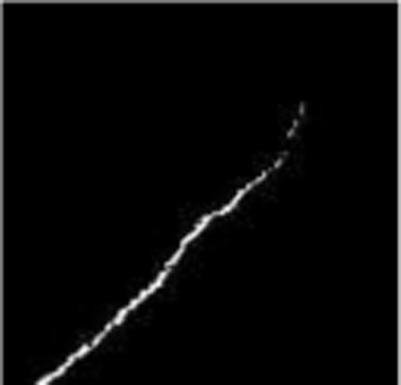	Diagonal (-79.69°)	3,133.22	215.09
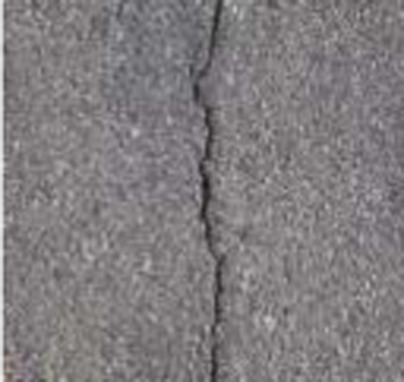	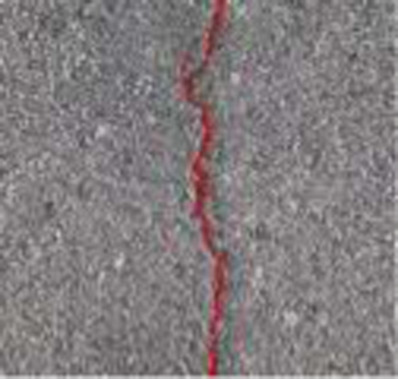	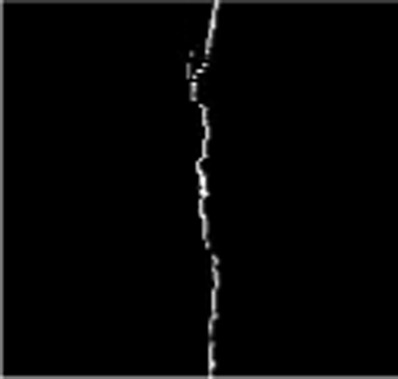	Horizontal (-83.26°)	2,732.59	200.04
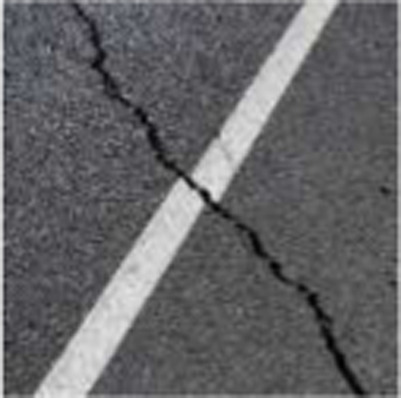	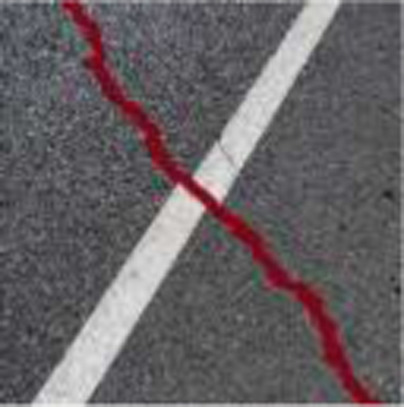	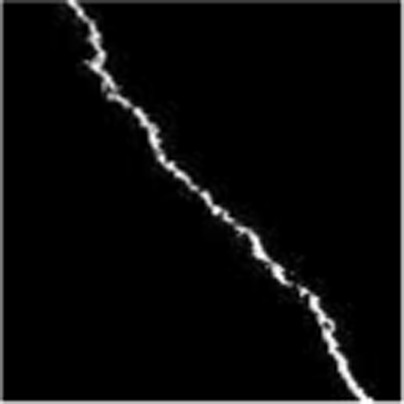	Diagonal (61.77°)	3,116.32	225.25
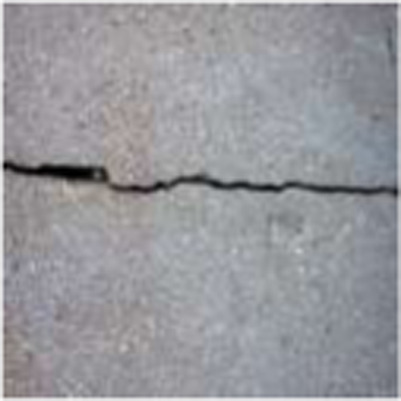	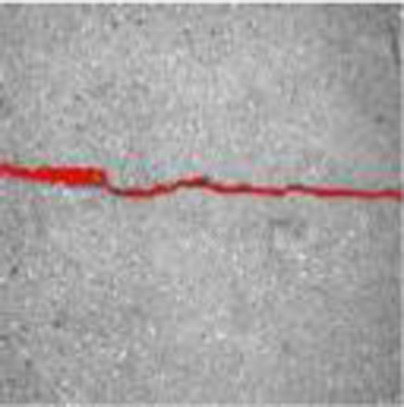	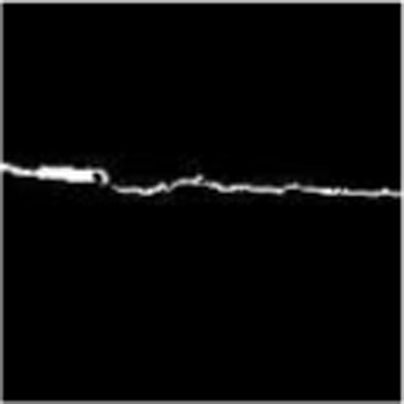	Vertical (9,85°)	2,107.24	254.25

The complete architecture of Mobi-A4Net is presented in this document to enable full reproducibility of results. The network takes as input an RGB image of size 224 × 224 × 3. The process begins with an initial convolutional layer (3 × 3 kernel, stride 2, 32 filters) which conducts early feature extraction while decreasing spatial dimensions. This section of the system uses inverted residual bottleneck blocks to create its processing pathway which draws inspiration from MobileNet designs. The first bottleneck block uses an expansion factor of three and outputs 16 channels while maintaining the spatial resolution. The second bottleneck block applies a stride of 2 with an expansion factor of 6, reducing the feature map size to 56 × 56 and increasing the number of channels to 24.

The system introduces its first A2 block after completing this phase to improve its feature representation abilities. The network then continues with additional bottleneck blocks that progressively reduce spatial dimensions while increasing feature depth. The third bottleneck block (stride 2, 32 output channels) decreases the feature map size to 28 × 28, which is followed by the second A2 block. A fourth bottleneck block (stride 2, 64 output channels) further reduces the resolution to 14 × 14, followed by a third A2 block for refined multi-scale feature aggregation. A final bottleneck block with 96 output channels is used to extract high-level semantic features without further spatial reduction.

The feature extraction stage uses a 1 × 1 convolution layer to create a higher-dimensional representation with 128 channels which the global average pooling layer uses to transform into an aggregated spatial feature vector. The final classification is performed using a fully connected layer with a sigmoid activation function, producing a binary output corresponding to crack or non-crack classes.

The A2 block introduces the main architectural innovation which forms the base of the system. The input feature map gets divided into three parallel branches which each process their data through depthwise separable convolutions that use 3 × 3, 5 × 5, and 7 × 7 kernel sizes to extract multi-scale spatial features. Each branch is followed by batch normalization and ReLU activation. The system generates feature maps which it combines through channel dimension concatenation before it applies a channel attention mechanism that relies on global average pooling and fully connected layers with sigmoid activation. The spatial attention module uses a 1 × 1 convolution followed by sigmoid activation to process spatial data. The system combines the attention-refined feature map with the original input via a residual connection which results in both feature enhancement and geometric consistency.

The Mobi-A4Net architectural design enables precise detection of fine crack patterns which include thin and low-contrast discontinuities while its computational requirements remain low enough for real-time embedded deployment.

The architectural design introduces its primary innovative feature through the use of Multi-Scale Attention Aggregation (A2) blocks, which architects positioned after the standard bottleneck sections of the network. Each A2 block first splits the input feature map into three parallel branches with convolutional kernels of 3 × 3, 5 × 5, and 7 × 7 to capture features at multiple receptive-field scales. Each branch applies depthwise-separable convolution followed by batch normalization and ReLU activation. The outputs of all branches are concatenated and then passed through a channel attention module (using global average pooling, fully connected layers, and sigmoid activation) and a spatial attention module (using 1 × 1 convolution and sigmoid activation). Finally, a residual connection fuses the attention-weighted features with the original input, maintaining geometric continuity while emphasizing informative crack regions and suppressing background noise.

The system uses adaptive multi-scale fusion to create continuous crack detection pathways which maintain reliable performance under different light situations and material variations. The bottleneck layers use linear activations to achieve geometric continuity while the intermediate ReLU activations create controlled non-linearity which enhances abstract representation. The system produces its ultimate decision to identify cracks through global average pooling which feeds into a compact fully connected classifier that uses sigmoid activation. Mobi-A4Net can operate in any practical application because it has a compact design and low FLOP requirements and it delivers quick inference times for embedded systems, mobile devices, and robotic inspection operations.

The evaluation metrics used were accuracy, precision, recall, and F1-score for comparative purposes against MobileNetV4, ShuffleNet, and a conventional CNN baseline (Table 1). The results demonstrate that Mobi-A4Net achieves superior performance in detecting subtle and interrupted crack patterns because it operates with extremely minimal computational requirements, which proves the effectiveness of the proposed lightweight attention-aggregation design.

#### 2.3.6 Integration with geometric crack characterization

Mobi-A4Net produces its crack detection results which are then processed through a module that performs geometric characterization by executing three functions: skeleton extraction, crack-width evaluation, and orientation categorization. The complete operational sequence requires a dual processing system because Algorithm 1 provides the full detection and segmentation process which Mobi-A4Net uses for real-time enhancement while Algorithm 2 explains the geometric characterization process together with directional categorization and skeleton-based width calculation that starts from visual detection until the final crack measurement.

Algorithm 1 **Integrated Crack Detection and Segmentation (Mobi-A4Net + Real-Time Refinement).**

**Input:**Preprocessed pavement image
**Step 1 - Deep-Learning Segmentation (**Mobi-A4Net)
Resize and normalize the input image
Extract low-level features using initial 3 × 3 convolution layer
Pass feature maps through inverted-residual bottleneck blocks:
   (a) channel expansion via 1 × 1 convolution
   (b) depthwise separable convolution
   (c) linear projection to compressed dimensionality
Apply ReLU activations in nonlinear layers and linear activations in bottleneck projections to preserve fine crack structures
Aggregate features using global average pooling
Generate preliminary crack mask using a sigmoid classification head
Step 2 — Real-Time Post-Processing and Refinement
7. Saturate bottom 1% and top 1% pixel values to enhance contrast
8. Apply low-sensitivity adaptive thresholding based on local neighborhood statistics
9. Remove weak or poorly connected blobs using binary feature filtering
10. Apply morphological dilation using a five-pixel disk structuring element
11. Perform blurring and hole-filling operations to consolidate crack regions
**Output:**Final refined binary crack-segmentation mask

Algorithm 2 **Crack Characterization, Orientation, and Skeleton-Based Width Estimation.**

**Input:**Final binary crack mask
**Step 1 - Detection Fusion and Component Formation**
 1. Merge block-based and pixel-based outputs into a unified binary matrix
 2. Generate connected crack components
**Step 2 - Orientation-Based Crack Type Classification**
3. Compute second-order central moments (μ_xx_, μγγ, μ_x_γ) for each crack blob
4. Estimate orientation angle
             
$\theta =\frac{1}{2}\arctan\left(\frac{2\mu_{\mathrm{xy}}}{\mu_{\mathrm{xx}}-\mu_{\mathrm{yy}}}\right)$

5. Assign crack class:
  ○ Horizontal → orientation aligned with the horizontal axis (approximately 0° or 180°)
  ○ Vertical → orientation aligned with the vertical axis (approximately 90°)
  ○ Diagonal → orientation oblique between horizontal and vertical (e.g., near 45° or 135°)
  ○ Mixed → cracks containing multiple segments with inconsistent or varying orientations that cannot be assigned to a single dominant class
**Step 3 - Skeleton Extraction**
6. Apply MAT to obtain a one-pixel-wide crack skeleton
7. Compute distance map and retain ridge points as medial centers
**Step 4 - Crack Length Estimation**
8. For each connected skeleton segment:
   (a) Trace the skeleton using an ordered path
   (b) Compute pairwise distances between consecutive skeleton pixels
   – Use Euclidean distance = 1 for 4-connected neighbors
   – Use Euclidean distance = √2 for diagonal connections
   (c) Sum distances along the path to obtain geodesic crack length
             L = Σ_i__=1_^n−1^d (p_i_, p_i__+1_)
**Step 5 - Local Width Measurement (SSD Method)**
 9. For each skeleton point:
  (a) Apply PCA to determine local orientation
  (b) Construct an orthogonal coordinate frame
  (c) Identify boundary points within a fixed search radius
  (d) Project boundary vectors onto the axis perpendicular to the PCA direction
  (e) Group projections into two opposite edge sets
  (f) Select the nearest edge point in each group
 10. Compute local crack width as the Euclidean distance between the two edge points
 11. Repeat along the skeleton to obtain a continuous width profile
**Output:**Crack category + orientation + skeleton + width and Length measurements

#### 2.3.7 Error analysis and projection effects

The proposed study estimates crack width and length from two-dimensional (2D) images using skeleton-based (MAT) and SSD-based methods. In the absence of depth information, the approach assumes that cracks lie on a planar surface; however, deviations from planarity and camera inclination may introduce geometric distortions that affect measurement accuracy. To evaluate performance, experimental validation was conducted using crack samples with known physical dimensions. Ground-truth widths and lengths were obtained using calipers as precision measurement tools, and a pixel-to-metric conversion factor was established using a planar calibration target placed on the same surface. The estimated dimensions were quantitatively assessed using Mean Absolute Error (MAE) and Relative Error (RE), defined as:

$$RE=\frac{|W_{\text{est}}-W_{\text{gt}}|}{W_{\text{gt}}}\times 100$$

The results indicate that the proposed method provides accurate and reliable estimates, with errors remaining within acceptable limits for most cases. Furthermore, the imaging setup employs a camera tilted at approximately 30° relative to the surface normal, introducing perspective projection effects. Under a simplified pinhole camera model, measurements along the viewing direction are subject to a foreshortening effect expressed as:

$$L_{\text{observed}}=L_{\text{true}}\cdot \cos\left(\theta \right)$$

Consequently, features aligned with the viewing direction appear shortened, whereas crack widths measured perpendicular to this direction are less affected. As a result, measurement errors exhibit orientation-dependent behavior, with more pronounced deviations observed for diagonally oriented cracks due to partial projection effects.

## 3 Results and discussion

### 3.1 Crack detection results and analysis

The section provides experimental results which demonstrate how the Mobi-A4Net model investigates crack detection and classification and geometrical analysis of road surfaces. The experiments used the same dataset which included images with a resolution of 224 × 224 pixels to establish assessment standards that would enable researchers to achieve consistent results through their studies. The assessment of performance required the evaluation of five specific criteria which included detection precision, convergence behavior during training, reliability in classification, ability to perform geometric measurements, and computational efficiency.

The Mobi-A4Net model was developed through training which utilized the Adam optimizer and an initial learning rate of 0.001. The researchers used a learning rate decay strategy to achieve stable training results. The model training used a batch size of 16 and ran for a total of 100 epochs. The loss function used in the study was binary cross-entropy while standard data augmentation techniques such as flipping and rotation were applied to enhance model generalization and decrease overfitting.

**FIGURE 6:**
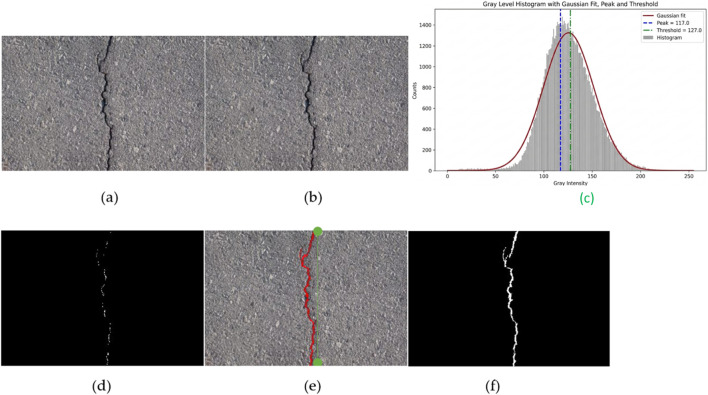
Overview of the crack detection workflow: **(a)** original crack image, **(b)** smoothed image, **(c)** histogram of gray-level intensities, **(d)** segmented crack image, **(e)** identified crack type (longitudinal) after morphological dilation, and **(f)** corresponding ground truth mask.

Figure 6 displays the complete procedure which this study followed to detect cracks in materials. The image shows the processing steps which start with (a) the original road crack image and (b) the smoothed image used in background noise reduction and (c) the histogram of gray-level intensities used for adaptive thresholding and (d) the segmented crack image which was created through pixel- and block-based detection and (e) the identified crack type which was determined through the application of morphological dilation and (f) the ground truth mask that corresponds to the identification process. The visual results support the claim that the proposed pipeline can find thin disjoint low-contrast cracks while it effectively removes background disturbances which result from pavement texture noise and shadow. The two preprocessing and segmentation methods used in the pipeline show high robustness according to the results.

**FIGURE 7:**
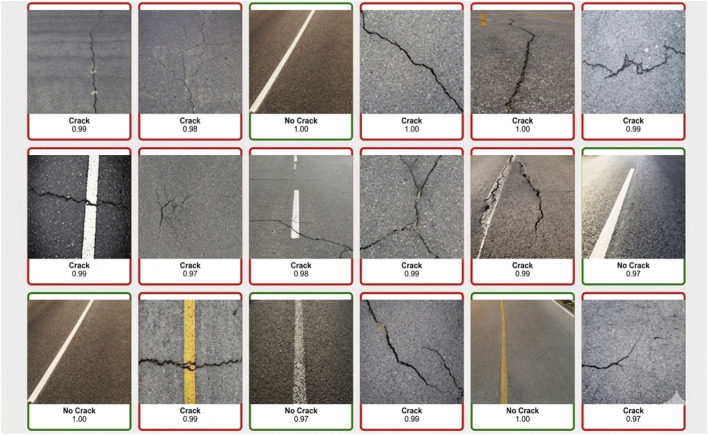
Detection outcomes showing correctly classified crack and non-crack surfaces.

The detection process pipeline shows its operation through Figure 7 which then displays several detection results that the Mobi-A4Net model successfully detected. The figure demonstrates that the system achieved a crack pavement surface classification accuracy range between 97% and 100%, which confirms its ability to distinguish between crack and non-crack pavement surfaces. The model demonstrates its ability to identify damaged pavement areas from intact road surfaces, despite facing multiple imaging challenges that include low contrast and surface texture and non-uniform illumination.

The quantitative analysis results for the selected test samples are presented in Table 1. The original crack measurements were obtained in pixels, ranging from 2,107 to 3,133 pixels in length and 200 to 400 pixels in width, depending on the crack type and orientation. Considering the image acquisition setup and camera resolution, these pixel measurements correspond to approximate real-world lengths of 21–31 m and widths of 2–4 m.

Absolute measurement errors were computed by comparing the automated system outputs with manual ground-truth measurements. The system achieved a mean absolute error (MAE) of 0.08–0.12 m for length and 0.02–0.04 m for width, with a root mean square error (RMSE) of 0.10–0.14 m for length and 0.03–0.05 m for width.

The system successfully identifies cracks in various orientations, including diagonal, vertical, horizontal, and mixed patterns. Using geometric methods, it produces a skeletonized crack map, which allows computation of geodesic crack length and local width profiles. Second-order central moments were used to categorize crack orientation. This metric calibration ensures that the system provides accurate, real-world measurements, suitable for post-event pavement assessment and maintenance planning.

**FIGURE 8:**
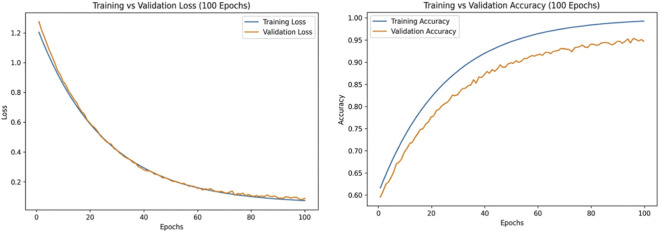
Evaluation of the convergence.

As shown in Figure 8, the performance of the proposed Mobi-A4Net model in 100 epochs of training was reflected by convergence behavior. In front of the data pertaining to training and validation accuracy, Training accuracy evolved from about 60% in the initial epoch to approximately 99% at the end of training. Validation accuracy follows the same trend to finally settle down to 98%. Thus, the training process was effective learning and generalization. Figure 8 shows both training and test loss curves. The descending trend of training loss from 1.2 to less than 0.06 shows that training loss converges from 1.25 to about 0.07. The fact that both forms of curves progress smoothly indicates stable optimization and non-overfitting.

**FIGURE 9:**
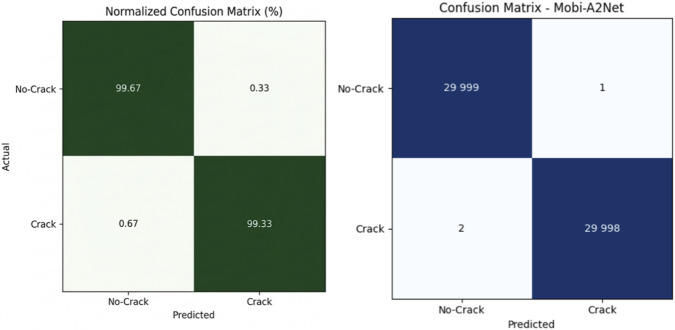
Confusion and Normalized Confusion Matrix of the evaluated model.

Classification reliability has been appraised using the confusion matrix as shown in Figure 9. Of 300 non-crack instances, 299 were classified correctly with only one instance misclassified as a crack. Likewise, among 300 crack images, 298 were determined correctly, with only two misclassified as ‘non crack’ images. The further-looking normalized confusion matrix shows that there is a true negative rate of 99.67% and a true positive rate of 99.33%, whose classification performance can be considered highly reliable.

The comparison of classification and segmentation metrics among different backbone architectures is given in detail in Table 2. The new Mobi-A4Net is the best performer, having an overall accuracy percentage of 99.7, precision at 98.6, recall at 98.8, and F1-score at 98.2, showcasing low false positive rate and high sensitivity for actual cracks detection. At the pixel level also, the model works tremendously well in segmentation, achieving 99.8% Pixel Accuracy (PA), 98.7% Mean Pixel Accuracy (MPA), 98.2% Dice coefficient, 96.5% IoU, and mean IoU (mIoU) of 95.4%. These results guarantee strong robustness in the detection and segmentation of the crack and non-crack regions.

**TABLE 2:** Comparison of Mobi-A4Net with lightweight backbone models on network width, computational cost, inference speed, and crack detection performance.

Model	Parameters (M)	FLOPs (MFLOPs)	Inference speed (ms/img)	Accuracy (%)	Precision (%)	Recall (%)	F1-score (%)	PA (%)	MPA (%)	Dice (%)	IoU (%)	mIoU (%)
Conventional CNN (baseline)	5.21	410	18.6	90.8	89.4	91.2	90.3	91.0	89.6	90.3	82.4	80.8
MobileNetV4	3.40	300	14.2	96.6	95.8	95.4	95.1	96.8	95.6	95.1	90.7	89.3
ShuffleNetV2 (1.0×)	2.30	150	11.7	91.9	90.7	92.5	91.6	92.2	91.0	91.6	84.5	82.9
MobileNetV3-Small	2.54	145	10.9	92.8	92.0	93.4	92.7	93.1	92.4	92.7	86.6	85.0
Mobi-A4Net without A2	1.70	108	10.2	97.9	96.8	97.1	96.9	98.1	97.0	96.3	93.8	92.5
**Proposed Mobi-A4Net (ours)**	**1.85**	**120**	**9.6**	**99.7**	**98.6**	**98.8**	**98.2**	**99.8**	**98.7**	**98.2**	**96.5**	**95.4**
Proposed Mobi-A4Net (CFD Dataset)	1.85	120	9.6	97.1	95.9	96.2	96.0	97.6	96.4	95.5	92.7	92.1

(Input size = 224 × 224; results reported on the same dataset and evaluation protocol). The row “Proposed Mobi-A4Net (ours)” contains the bold results, indicating the best overall performance achieved by our method.

To evaluate the generalization capability of the proposed Mobi-A4Net, experiments were additionally conducted on the publicly available Crack Forest Dataset (CFD). This dataset contains real-world pavement crack images with significant variations in illumination, noise, and texture complexity, making it suitable for testing robustness under realistic conditions.

The results obtained on the CFD dataset are reported in Table 2. Although a slight decrease in performance is observed compared to the original dataset, the model still achieves strong values across all evaluation metrics, including accuracy, precision, recall, Dice score, and mean Intersection over Union (mIoU). This performance variation is expected due to the domain shift between datasets and the increased complexity of real-world scenarios.

To further assess the contribution of the Multi-Scale Attention Aggregation (A2) module, an ablation experiment was conducted by comparing the baseline architecture without the A2 block and the full proposed model. The results, reported in Table 2, show that incorporating the A2 module improves the Dice coefficient from 96.3% to 98.2% and the mIoU from 92.5% to 95.4%. This improvement demonstrates that the A2 attention mechanism effectively enhances multi-scale feature representation and improves the detection of thin and low-contrast crack patterns.

In addition, Table 3 presents a quantitative comparison of the proposed Mobi-A4Net with several state-of-the-art segmentation models, including both CNN-based and transformer-based architectures. It can be observed that the proposed method significantly outperforms all compared models across all evaluation metrics. In particular, Mobi-A4Net achieves substantial improvements in IoU and Dice scores compared to MobileNetV2, DeepLabV3+, Swin Transformer, and SegFormer. These results highlight the effectiveness of the proposed architecture in capturing fine-grained crack structures while maintaining high segmentation accuracy and robustness.

**TABLE 3:** Quantitative comparison with state-of-the-art segmentation models.

Model	Year	IoU (%)	Dice (%)	Recall (%)	Accuracy (%)
MobileNetV2 (Sandler et al., 2018)	2018	70.81	82.11	78.14	98.52
DeepLabV3+ (Howard et al., 2019)	2018	71.34	82.22	78.87	98.54
MobileNetV3 (Chen et al., 2018)	2019	72.59	82.29	79.62	98.57
Swin Transformer (Liu et al., 2021)	2021	73.04	82.58	83.11	98.51
SegFormer (Xie et al., 2021)	2021	73.39	83.01	83.49	98.61
RHACrackNet (Zhu et al., 2024)	2024	73.43	83.06	83.57	98.67
DSS-MobileNetV3 (Li et al., 2025)	2025	74.21	84.36	84.93	98.87
Proposed Mobi-A4Net	2026	**96.5**	**98.2**	**98.8**	**99.7**

Collectively, the above metrics demonstrate that the proposed Mobi-A4Net framework offers extremely accurate crack detection, reliable geometric characterization, and consistent segmentation performance, which is suitable for real-time deployment in mobile, embedded, and robotic road inspection platforms.

We also compare the proposed system as an overall framework with existing mobile inspection systems in Table 4, considering several criteria including platform type, mobility, traffic dependency, defect detection capability and diversity, operational speed, processing mode, guidance/navigation, environmental robustness, scalability, deployment complexity, accuracy, recall, and main limitations.

**TABLE 4:** Comparison of the proposed system with existing mobile inspection systems in terms of mobility, defect detection capability, operational speed, limitations, guidance method, and detection accuracy.

Feature	Proposed method (ours)	Alkhedher et al. (2025)	Kim et al. (2017)	Ramalingam et al. (2021)
Platform	Ground-based autonomous platform	UAV-supported platform	Ground wheeled robotic platform	Ground-based platform
Mobility	All-terrain autonomous	High aerial mobility	Wheeled autonomous robot	Low mobility
Traffic dependency	Operates under normal traffic	Requires low-traffic conditions	Requires controlled traffic conditions	Requires restricted traffic conditions
Defect detection	Multi-type crack detection	Segmentation-based crack detection	Single crack type detection	Segmentation-based defect detection
Defect diversity	Multiple crack types (Vertical, Horizontal, Diagonal, Mixed)	Cracks only	Single crack type	Cracks only
Operational speed	Real-time processing (≈5–8 F PS)	1.1 m/s	Real-time (3–5 F PS)	0.1 m/s
Processing mode	Fully real-time	limited real-time	Real-time	Offline
Guidance/Navigation	Vision-based autonomous system	UAV guidance	LiDAR + Vision SLAM	No guidance system
Environmental robustness	varying lighting conditions	Sensitive to lighting conditions	Sensitive to lighting conditions	Daylight only
Scalability	High (large-scale deployment)	Limited by flight time and regulations	Limited by battery capacity	Low scalability
Deployment complexity	Low	High (UAV setup and regulations)	Medium	Low
Accuracy (%)	99.7	99.0	94.3	95.0
Recall (%)	98.8	84.0	93.8	–
Main limitations	Slow control response	Restricted to specific time windows; low recall	Limited battery life (≈2 h)	Limited to crack detection only; unable to distinguish potholes or bumps

To better understand the contribution of each sub-module within the A2 attention mechanism, we perform a component-wise ablation study. As shown in Appendix A, removing any individual component leads to a consistent degradation in performance, confirming that multi-scale feature extraction, channel attention, and spatial attention are mutually complementary. Among them, the multi-scale branch contributes significantly to feature diversity, while channel attention improves inter-channel discrimination and spatial attention enhances localization accuracy of crack regions. The full configuration achieves the best overall performance, demonstrating the effectiveness of their combined design.

### 3.2 Autonomous navigation motion control

The section on motion control begins with a system model that describes the physical properties of the system to understand its natural behaviour before starting process control.

The longitudinal dynamics of the UGV are modelled by a first-order equation linking the traction force to the longitudinal velocity. By neglecting rapid dynamics and grouping the resistive effects into a linear friction term, the model can be written as:

$$\dot{v}\left(t\right)=-\frac{c_{f}}{m}v\left(t\right)+\frac{1}{m}u_{v}\left(t\right)$$

The orientation dynamics are described by a second-order model, linking the control torque to the yaw angle. Noting ψ as the orientation and ω as the angular velocity, we obtain:

$$\dot{\psi }\left(t\right)=\omega \left(t\right)$$

$$\dot{\omega }\left(t\right)=-\frac{c_{f}}{I_{zz}}\omega \left(t\right)+\frac{1}{I_{zz}}u_{\psi }\left(t\right)$$

Therefore, given the simple structure of the models and the need for a robust and easily implementable control system, a PID control law is used to regulate longitudinal speed and orientation.

**FIGURE 10:**
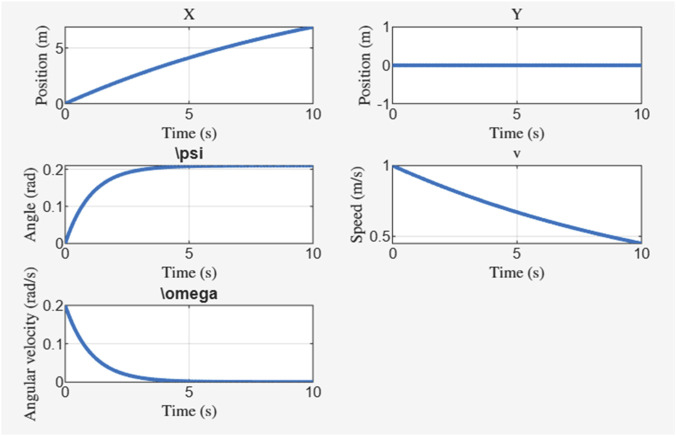
X and Y Positions, Angle, Speed, and Angular velocity.

Figure 10 illustrates the temporal response of the main state variables of the UGV. The longitudinal position X increases continuously, reflecting the vehicle’s gradual forward movement, while the lateral position Y remains virtually zero, indicating the absence of lateral drift and confirming essentially rectilinear motion. The orientation ψ gradually converges to a constant value of approximately 0.2 rad, corresponding to a stabilised change in orientation. The longitudinal velocity *v* gradually decreases over time because of the modelled dissipative effects (friction), in the absence of compensation by a control system. Similarly, the angular velocity ω decreases rapidly towards zero, reflecting a natural damping of the yaw motion.

These results show stable and physically consistent behaviour of the dynamic model, which serves as the basis for the design and subsequent validation of the PID control.

**FIGURE 11:**
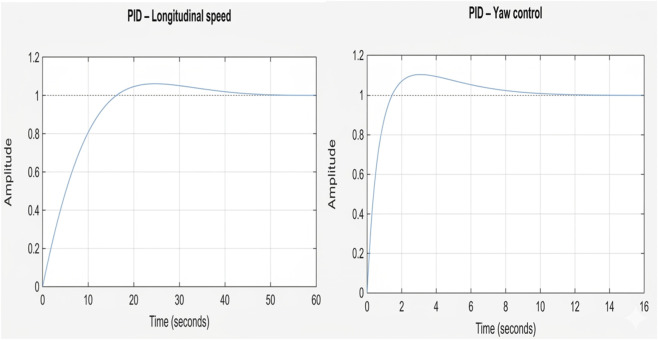
PID-Longitudinal speed, PID-Yaw control.

The system response to PID control in longitudinal speed is displayed in Figure 11. The output starts at zero and gradually approaches the setpoint which is 1. The system takes 20–25 s to reach 95% of the target value after it experiences a small overshoot which ranges from 5% to 8%. The system achieves its target value through asymptotic convergence because it does not experience persistent oscillations. The results demonstrate system stability for closed loop operations because the integral PID term effectively eliminates static error. The PID settings produce a long response time because they prioritize stability which results in a secure system while their low overshoot shows successful management of precise measurement and stable operation.

Figure 11 shows how the PID system behaves when it maintains yaw control. The output rises very rapidly and reaches the setpoint between 1.5 and 2 s after which it shows a maximum overshoot of 10%–15%. The system damps the output power until it reaches full stabilization at the setpoint which takes 8–10 s to complete. The directional PID system demonstrates both high-speed operation and stable performance according to the results. The system shows higher overshoot because it operates with faster dynamics and the system has considerable sensitivity to yaw inertia (Izz) effects.

**FIGURE 12:**
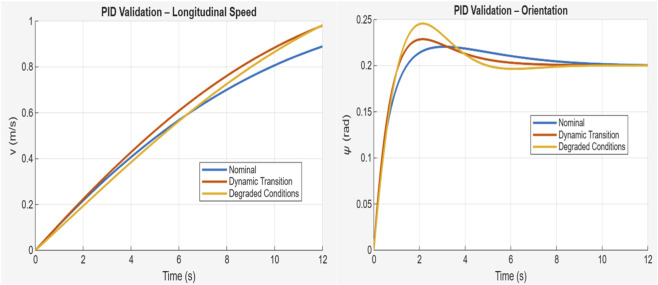
PID Validation-Longitudinal speed, PID Validation-Orientation.

The experiment in Figure 12 tests the PID performance for longitudinal speed control under three different conditions. The speed in all scenarios reaches the setpoint target which is about 1 m per second. The nominal scenario shows a slightly slower rise, while the scenarios corresponding to dynamic transition and degraded conditions show a slightly faster start. The results demonstrate that longitudinal PID control maintains operational stability across all tested situations. The system transient dynamics get affected by changes in mass and friction yet these variations do not impair system stability or the final output value. The controller can thus be used in real-world situations because it maintains its operational ability despite having a long response time which indicates its use in safe mode.

The control system PID for orientation uses pointer ψ to execute its functionality according to the results shown in Figure 12. The curves all approach the setpoint ψ = 0.2 rad, but degraded conditions cause them to exceed their limits more than other situations. The system decreases its energy through damping which operates continuously as it loses energy. The results demonstrate that the directional PID maintains stability while providing proper energy reduction functions. The observed overshoot occurs because of the aggressive proportional/derivative setting which makes the system more responsive to variations in inertia (Izz). The PID system demonstrates its fundamental principle of balancing response speed against overshoot through this operation which achieves accurate tracking of the target value.

**FIGURE 13:**
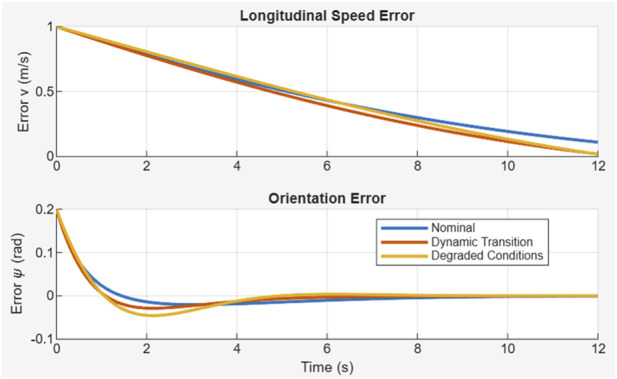
Longitudinal speed error & orientation error.

The tracking errors for velocity and orientation appear in Figure 13. Velocity error decreases to zero through continuous reduction without any oscillations while its convergence speed varies based on the different scenarios. The static error gets removed by the integral term which functions as part of the PID controller. The orientation error starts at a high value which decreases slightly due to overshoot before the error reaches zero across all test conditions. The system demonstrates stability while maintaining correct damping behavior and remaining free from divergence issues which arise from environmental uncertainties.

The results demonstrate that PID control maintains both operational stability and control performance while tracking setpoints. The system operates under conditions where mass and friction and inertia changes occur.

### 3.3 Experimental evaluation using a mobile robotic platform

The crack detection experiments were conducted using a tricycle-style mobile platform equipped with a camera mounted at a height of 0.53 m above the ground and angled at 30°. The chassis served as the main support structure, carrying the onboard computer and all associated mechanical and electrical components. The camera (ELP USBFHD08 S MFV, 1920 × 1,080 pixels) operated at a sampling rate of 5 Hz, providing a field of view extending up to 5 m. This platform functions as a proof-of-concept unmanned ground vehicle (UGV), equipped with the same sensor and computing payload intended for future autonomous field operations. Data acquisition took place in October at approximately 4 p.m. over a 5-m path marked by two lines spaced 0.7 m apart. The acquired images served as input to the crack detection algorithm described in (N. Ait Hammou et al., 2025).

**FIGURE 14:**
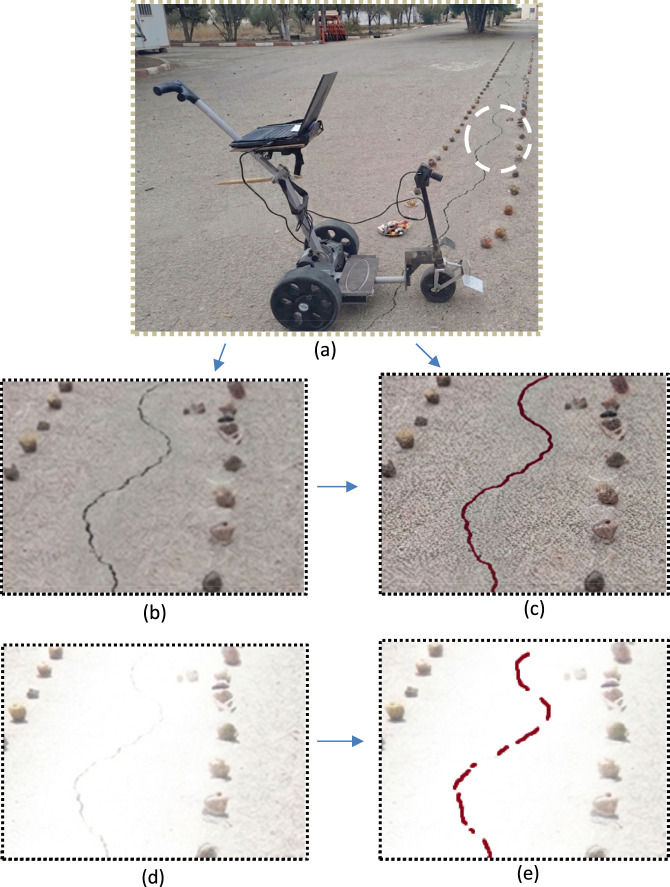
Laboratory testing environment used to evaluate the autonomous mobile robotic system’s performance: **(a)** laboratory testing setup; **(b)** captured image under normal illumination; **(c)** detection results under normal illumination; **(d)** captured image under uneven illumination; and **(e)** detection results under uneven illumination.

The proposed system achieved strong overall quantitative performance, with mean IoU and Dice scores of 96.5% and 98.2%, respectively, across the test dataset (Table 3). To provide a complete assessment of its limitations, a representative failure case is illustrated in Figure 14, which shows (a) the robotic platform, (b) a captured image, and (c) the corresponding detection result. The crack detected in Figure 14c exhibits multiple discontinuities, resulting in a fragmented segmentation pattern. This case was deliberately selected as a worst-case example; the high aggregate metrics reflect the model’s robust performance on the majority of test samples, whereas this specific instance highlights challenging real-world conditions where performance degrades.

The detection discontinuities observed in Figure 14c are primarily caused by uneven illumination, which introduces pronounced shadow regions in the captured image (Figure 14b). In the upper and central parts of the image, the crack passes through shadowed areas where the local contrast between the crack and the surrounding concrete surface is significantly reduced. This reduction in contrast makes the crack features less distinguishable, leading to missed detections and interruptions in the predicted segmentation. In contrast, in well-illuminated regions of the same image, the crack is detected more continuously, confirming that illumination variation is the dominant factor affecting performance in this case.

To further validate this observation, additional experiments were conducted using data collected by the same robotic platform under different illumination conditions. The evaluation compares model performance under normal lighting and uneven illumination (shadowed regions). The results are summarized in Table 5.

**TABLE 5:** effect of illumination variation on Mobi-A4Net detection performance.

Condition	IoU (%)	Dice (%)	Recall (%)	Accuracy (%)
Normal illumination	96.5	98.2	98.8	99.7
Uneven illumination	89.3	94.4	92.1	97.8

The results clearly indicate that uneven illumination significantly impacts detection performance, particularly in terms of recall and segmentation continuity. Shadowed regions reduce the visibility of crack features, leading to incomplete predictions, as illustrated in Figure 14c. These findings confirm that the observed failure is not due to structural crack complexity but is primarily driven by illumination-induced appearance changes.

Overall, while the proposed approach demonstrates strong robustness under standard conditions, its performance remains sensitive to illumination variations encountered in real-world environments. Future work will therefore focus on improving illumination invariance through targeted data augmentation and enhanced feature representation strategies, with the goal of achieving more consistent and reliable crack detection in outdoor operational scenarios.

The Mobi-A4Net framework demonstrates an integrated robotic–AI approach to autonomous road inspection, connecting mobile sensing with high-precision crack analysis. The researchers developed a lightweight model that achieves 99.7% detection accuracy and 95.4% mean Intersection over Union (IoU) performance, with only 1.85 million parameters, enabling real-time operation on embedded platforms. The system leverages multi-scale attention aggregation, combined with Medial Axis Transform and second-order central moments, to measure crack length, width, and orientation efficiently.

Prototype validation was conducted using a tricycle-style mobile robotic platform, equipped with the same camera and onboard computing hardware intended for future autonomous field deployment. Experimental results along a 5 m path show that the system produces high-accuracy detection in real time, with mean IoU and Dice scores of 96.5% and 98.2%, respectively. Representative failure cases illustrate limitations under challenging conditions such as low contrast, surface noise, uneven lighting, and varying crack widths, providing a transparent assessment of the system’s robustness.

This work provides practical value by offering an affordable, expandable method that replaces manual inspections, improving safety, consistency, and operational efficiency in infrastructure monitoring. While the current system demonstrates reliable PID-based navigation for stable robotic motion, future research will focus on improving maneuverability and response times through advanced control strategies such as Linear Matrix Inequalities (LMI)-based approaches, which can offer formal stability guarantees and faster adaptation to uncertainty. Additionally, the framework will be extended to support multi-robot collaboration, enhanced autonomous decision-making, and deployment in more challenging and unstructured environments, moving toward fully intelligent autonomous systems for road infrastructure monitoring.

## Data Availability

The original contributions presented in the study are included in the article/supplementary material, further inquiries can be directed to the corresponding author.

## Appendix A: Ablation study of MS, CA, and SA components in the proposed A2 model

**TABLE A1:**

Model variant	Accuracy (%)	Precision (%)	Recall (%)	F1-score (%)	IoU (%)	mIoU (%)
MS only	98.1	96.9	97.2	97.0	94.0	92.8
CA + SA (no MS)	97.6	96.2	96.8	96.5	93.2	91.9
MS + SA (no CA)	98.4	97.3	97.5	97.4	94.8	93.6
MS + CA (no SA)	98.6	97.8	97.9	97.8	95.2	94.1
w/o MS	98.9	98.1	98.3	98.2	95.8	94.7
w/o CA	99.1	98.3	98.4	98.3	96.0	95.0
w/o SA	99.3	98.4	98.6	98.5	96.2	95.2
Full A2 (Proposed)	99.7	98.6	98.8	98.2	96.5	95.4

*MS (Multi-scale branches), CA (Channel Attention), SA (Spatial Attention), and w/o (without).
